# Practicing Medicine Wisely: Routine Use of Urine Legionella in Suspicious Pneumonia - Are we Overdoing?

**DOI:** 10.31729/jnma.8621

**Published:** 2024-06-30

**Authors:** Swotantra Gautam, Aakash Neupane, Luis Isea Mercado, Phuong Nguyen, Suzit Bhusal

**Affiliations:** 1 Department of Internal Medicine, Advent Health Orlando, Florida, USA; 2 Department of Internal Medicine, B.P. Koirala Institute of Health Sciences, Dharan, Sunsari, Nepal; 3 Research and Development Unit, National Trauma Centre, Bir Hospital, Kathmandu, Nepal

**Keywords:** *Legionella species*, *Legionnaires disease*, *urinary antigen testing*, *viewpoint*

## Abstract

Legionella spp. is an underreported cause of Community Acquired pneumonia that affects significant population specially in urban areas and its prevalence is on an increasing trend. The routine practice of testing for urinary antigen of Legionella in all suspected cases of pneumonia is prevalent is resource rich western countries. Although several studies have shown no distinct advantage of performing routine urinary antigen testing, this practice continues to be preferred by clinicians. In this viewpoint, we have discussed the advantages and disadvantages of urinary antigen testing, its relevance in current practice and its impact on clinical outcomes.

## INTRODUCTION

*Legionella pneumophila* was first identified as a cause of severe community acquired pneumonia (CAP) during the 1976 American Legion convention in Philadelphia. It is a significant cause of CAP, causing 2-3.4% of cases in the United States.^1^ The CDC estimates that there are approximately 5,000 cases of Legionnaires' disease reported annually in the United States which has increased by almost ninefold since 2000. In the context of Nepal, although legionella species has been identified in shallow depth well water and other public water systems,^2^ legionnaires disease is under reported. This may be due to unavailability of testing facilities in Nepal.

## IS ROUTINE SCREENING NECESSARY?

Various diagnostic methods are available for Legionella detection including culture, urine antigen detection, serological assessments, direct fluorescent antibody staining or immunohistochemistry, and polymerase chain reaction (PCR) testing.^3^ Among the battery of tests available, Urinary antigen testing (UAT) accounts for a substantial proportion of diagnostic tools employed for confirming Legionnaires' disease (LD) cases. Several commercially available urinary antigen tests for *L. pneumophila* offer a rapid diagnosis with high sensitivity and specificity. Current Infectious Diseases Society of America/American Thoracic Society Legionella Pneumonia (IDSA/ATS) guidelines only recommend performing a Legionella urinary antigen test in patients with severe CAP and those with epidemiological risk factors for Legionella.^4^ In a randomized trial encompassing 177 patients, where participants were allocated to either pathogen-directed treatment (targeted treatment) determined by urinary antigen testing outcomes for *S. pneumoniae* and *Legionella* or empirical guideline-directed treatment. The study findings did not reveal any significant differences in terms of mortality, clinical relapse, admission to the intensive care unit (ICU), duration of hospital stay, or length of antibiotic regimen.^5^ In another trial involving 262 participants, Patients who underwent pathogen-directed therapy exhibited comparable clinical outcomes to those who received empirical, guideline-directed therapy.^6^

In contrast, there are studies such as one demonstrating a statistically significant reduction (57%) in the odds of in-hospital mortality for patients who underwent pneumococcal and Legionella urinary antigen testing when compared to patients who were not tested.^7^ Similarly, another trial led by Uematsu et al. demonstrated a 25% decrease in the odds of 30-day mortality among patients who received urinary antigen tests; however, this did not influence the length of hospital stays.^8^

Randomized trials have not succeeded in demonstrating a clear advantage of urinary antigen testing for identifying *S. pneumoniae* and *Legionella* infections. Furthermore, concerns have been raised regarding the potential for narrowing treatment choices solely based on positive urinary antigen tests, which might lead to an elevated risk of clinical relapse. To address these uncertainties, it becomes imperative to undertake studies designed to minimize any confounding factors and optimize the potential benefits derived from these swift diagnostic tests.^5^

One approach can be to adopt a modified design that involves a markedly shorter initial treatment duration, potentially consisting of just a single dose. Subsequently, the results of urine antigen tests could serve as a guiding factor for either continuing with an empirical antimicrobial regimen or transitioning to targeted therapy. Such a method would not only allow for a more refined treatment strategy but could also mitigate the associated risks and uncertainties.

## IS LEGIONELLA URINARY ANTIGEN TEST ENOUGH?

As outlined in a comprehensive pooled analysis, Legionella urinary antigen testings' utility is more inclined towards confirming rather than ruling out the disease. In a clinical context, a positive urinary antigen test result serves as a strong indicator of Legionellosis, yet a negative result does not definitively exclude the presence of the disease. Intriguingly, research indicates that 26% of patients with confirmed Legionellosis yield a negative urinary antigen test result. Notably, the current urinary antigen tests appear to lack effectiveness in detecting serogroups other than type 1.^3^

Similarly, a study conducted in central Texas highlighted a reduced occurrence of Legionella pneumonia cases identified through urine antigen testing. This is largely attributed to the implementation of guideline-congruent antibiotic treatment, which inherently covers *Legionella* cases empirically. Consequently, it is speculated that testing in regions with low disease prevalence may not significantly impact outcomes and could potentially lack cost-effectiveness.^9^

The sensitivity of the UAT varies depending on the serogroup of Legionella, the severity of the disease, and the time since the onset of symptoms.^10^ For example, the sensitivity of the UAT for L. pneumophila serogroup 1, the most common serogroup that causes Legionnaires' disease, is about 70% in patients with severe pneumonia, but only about 40% in patients with mild pneumonia.^11^ The UAT also has some disadvantages, such as the inability to detect Legionella species other than L. pneumophila serogroup 1 and the inability to diagnose relapse or reinfection due to the persistence of antigen excretion.^12^ For these reasons, it is important to consider adding additional testing to enhance the likelihood of diagnosing Legionnaires' disease if the UAT is positive. Some of the additional tests that can be used include direct fluorescent antibody test, serology and culture. Numerous research teams have evaluated the potential advantages of an integrated methodology for identifying Legionella. The recent strategy involves moving away from the risk factor-based approach when determining testing indications. Instead, the focus is on adopting alternative methods, such as assessing patient types and clinical scenarios that would derive the greatest advantage from microbiological diagnoses.^13^ Their findings consistently demonstrate enhancements in both diagnostic precision and accuracy through the utilization of multiple complementary assays. For instance, employing a blend of PCR, culture, UAT (urinary antigen testing), and/or IFA (immunofluorescence assay) has led to notable improvements in overall diagnostic sensitivity and specificity.^14^

## COST/BENIFIT ANALYSIS

Enhancing the effectiveness of testing holds considerable significance, as research indicates that the expenses associated with each positive outcome from Legionella UAT (urinary antigen testing) can range from USD $850 to $12,640, contingent on the local prevalence of Legionella. In regions with low occurrences of Legionella, such as the southern and Pacific regions of the United States, routine testing for Legionella might not yield cost-efficient outcomes. From a perspective centered on cost-effectiveness, the infrequent occurrence of positive results in UAT testing leads to a substantial financial burden for each positive case, highlighting the urgency of optimizing the testing process.^15^ As an illustration, a comprehensive investigation conducted in central Texas disclosed a mere 0.3% (17 out of 5807) incidence of positive Legionella antigen among patients admitted with pneumonia. Consequently, the expenditure incurred to diagnose a solitary instance of Legionella-induced pneumonia amounted to an astounding USD $12,6409.

In various retrospective studies conducted globally, adherence to proper guidelines and prescribing broad-spectrum antibiotics with coverage of legionella have good outcomes as compared to guideline noncompliance.^7^ One institution saw a significant decline in the number of tests ordered in 2010 after the publication and dissemination of information regarding Legionella, including some of the findings shown in this study. It was reasoned that this decline in testing may have been a result of improved physician education on the low incidence of Legionella in our region and the proper guidelines regarding testing in severe CAP.

## CONCLUSIONS

It is important to note that although numerous studies have highlighted the potential benefits, routine Legionella testing in regions with low occurrence of legionella and for all cases of community-acquired pneumonia (CAP) might not necessarily yield any advantage in terms of reducing mortality or morbidity. Furthermore, it could be an added financial burden. However, as reductions in mortality have been linked to these diagnostic assessments in extensive observational studies, we agree with the IDSA/ATS guidelines to conduct testing in individuals experiencing severe disease. The rapid rise of Legionella infections over the last ten years in the United States accentuates the critical nature of this diagnosis, particularly for critically ill patients. Thus, more quality trials exploring the potential of Urine antigen in the context of reducing mortality/morbidity and financial burden are overdue.
